# Quantification of Ultra-Trace Lead in Water After Preconcentration on Nano-Titanium Oxide Using the Slurry Sampling ETAAS Method

**DOI:** 10.3390/toxics13080610

**Published:** 2025-07-22

**Authors:** Lucia Nemček, Ingrid Hagarová

**Affiliations:** Institute of Laboratory Research on Geomaterials, Faculty of Natural Sciences, Comenius University in Bratislava, Mlynská dolina, Ilkovičova 6, 842 15 Bratislava, Slovakia; ingrid.hagarova@uniba.sk

**Keywords:** ultra-trace lead, nano-titanium oxide, slurry sampling, electrothermal atomic absorption spectrometry, dispersive micro solid-phase extraction, water analysis

## Abstract

A simple and efficient dispersive micro solid-phase extraction (DMSPE) method using nano-TiO_2_ as a sorbent was developed for the separation and preconcentration of (ultra) trace levels of lead in water samples prior to quantification by electrothermal atomic absorption spectrometry (ETAAS). Key experimental parameters affecting the DMSPE process, including pH, ionic strength, sorbent dosage, and preconcentration factor, were optimized. The optimized method demonstrated a preconcentration factor of 20, a relative standard deviation below 4.5%, and a detection limit of 0.11 µg/L. The procedure was validated using certified reference material (CRM TM-25.5) and applied to real water samples from a lake, a residential well, and industrial wastewater. Satisfactory recoveries (89–103%) confirmed the reliability of the method for the determination of low lead concentrations in complex matrices.

## 1. Introduction

Lead is a naturally occurring metal widely used in various industries, though its use is being gradually restricted due to well-documented health and environmental risks. With no known biological function in the human body, lead is toxic even at low concentrations. As a cumulative toxin, it accumulates in the bones, liver, kidneys, and nervous system, making long-term exposure to even minimal amounts potentially harmful [1]. Chronic exposure to lead can cause nervous system damage, cognitive impairment, kidney dysfunction, high blood pressure, and anemia [2]. Therefore, the ability to reliably quantify lead at both trace and ultra-trace levels across various matrices is crucial, with water being among the most frequently analyzed.

Lead concentrations in natural waters typically range from 2 to 10 µg/L [3]. While these levels are generally considered low, human activities can significantly increase lead contamination, highlighting the importance of regular monitoring. This is particularly important for drinking water due to its direct impact on human health. To protect public health, organizations worldwide have established limits for acceptable lead concentrations in drinking water. The World Health Organization (WHO) recommends a guideline value of 10 µg/L (0.01 mg/L) [4], a threshold that has been adopted by many countries, including Slovakia and other members of the European Union [5,6]. Some nations have implemented even stricter standards; for example, in March 2019, Canada lowered its maximum acceptable concentration for lead in drinking water to 5 µg/L (0.005 mg/L) [7], reflecting evolving scientific evidence and a stronger emphasis on safety.

In addition to drinking water, monitoring lead concentrations in natural and industrial water sources is also essential. Surface waters such as lakes may be affected by atmospheric deposition, agricultural runoff, or nearby industrial activity, all of which can contribute to elevated lead levels. Although these waters are not typically used directly for consumption, they can serve as sources for drinking water supply or recreational use, making contamination a concern. Industrial processes, such as those in petrochemical plants, generate wastewater that may contain a variety of pollutants, including heavy metals like lead. This wastewater is routed to specialized wastewater treatment plants, where advanced purification technologies are employed. Despite the high effectiveness of these systems, trace and ultra-trace concentrations of lead can remain, requiring sensitive analytical techniques for accurate monitoring. Given the complexity of such matrices and the often very low concentrations of lead, reliable determination depends not only on sensitive instrumentation but also on efficient sample preparation.

To meet the demands of such diverse and often complex water matrices, spectrometric methods capable of detecting ultra-trace concentrations of lead are necessary for effective environmental surveillance and risk assessment. Among them, inductively coupled plasma mass spectrometry (ICP-MS), with its exceptional sensitivity and multielement capabilities, is widely regarded as the most suitable technique for the reliable determination of ultra-trace lead. However, its high operational costs and limited availability in many laboratories often necessitate the use of alternative approaches. In such cases, particularly when single-analyte determination is required, other spectrometric techniques such as flame atomic absorption spectrometry (FAAS) or electrothermal atomic absorption spectrometry (ETAAS) are commonly employed. ETAAS, in particular, is known for its excellent selectivity and sensitivity, and when combined with an effective separation and preconcentration step, it enables accurate quantification of lead even at low microgram per liter (µg/L) or nanogram per liter (ng/L) levels across a range of water types, including drinking water, surface water, and treated industrial effluents.

In recent years, various extraction techniques have been employed to enhance detection by concentrating the analyte, thereby improving the sensitivity of detection techniques. Among these, solid-phase extraction (SPE) using nanomaterials of diverse origins, compositions, and structures has proven to be particularly effective. When solid nanomaterials are used as sorbents, the dispersive mode of SPE, referred to as dispersive solid-phase extraction (DSPE), is typically preferred. In this approach, the sorbent is directly dispersed into the sample solution, significantly increasing the surface contact between the analyte and sorbent, which is particularly advantageous given the high surface area and reactivity of nanomaterials. When only small quantities of sorbent are used (typically a few to several tens of milligrams), the technique is classified as dispersive micro solid-phase extraction (DMSPE). Among the nanomaterials applied in this context, metal-containing nanoparticles are frequently employed as inorganic sorbents. This category includes a wide variety of materials, such as pure metals (e.g., Au, Ag, Cu, Fe), metal oxides (e.g., TiO_2_, ZrO_2_, ZnO, CuO, Al_2_O_3_, Fe_x_O_y_), and other metal-based nanoparticles (e.g., CdSe/ZnS quantum dots) [8].

The present study focuses on developing a simple and effective method for determining (ultra)trace levels of lead in various aqueous samples, using DMSPE with nano-TiO_2_ as the sorbent, followed by direct injection of the resulting nanoparticle slurry (containing the preconcentrated lead) into the graphite furnace of ETAAS. The method is designed to combine the advantages of nanomaterial-based micro solid-phase extraction with the high sensitivity of ETAAS, enabling reliable lead quantification across different water types. Special attention is given to optimizing key parameters influencing extraction efficiency and detection performance. The applicability of the method is demonstrated on real water samples, including drinking water from a well, surface water from a natural lake, and treated industrial wastewater from a petrochemical facility.

## 2. Materials and Methods

### 2.1. Instrumentation

Lead concentrations were determined using electrothermal atomic absorption spectrometry (ETAAS) with a Perkin-Elmer 4100ZL atomic absorption spectrometer (Überlingen, Germany) equipped with a transversely heated graphite atomizer (THGA) and AS-70 autosampler. The system employed Zeeman-effect background correction and pyrolytically coated graphite tubes with integrated L’vov platforms (Perkin-Elmer). A hollow cathode lamp for lead (Perkin-Elmer) operating at 10 mA served as the radiation source. The wavelength selected for measurement was 283.3 nm with a spectral bandwidth of 0.7 nm. Absorbance values were evaluated by measuring the total area of the signal peak (integrated absorbance). Argon was used as a purge gas at a flow rate of 250 mL/min. A 20 µL aliquot of the sample solution was injected into the graphite furnace, followed by 10 µL of a 2% (w/v) NH_4_H_2_PO_4_ chemical modifier solution. Calibration solutions for lead (10–80 µg/L) were prepared in 0.2% (v/v) HNO_3_. The temperature program used for lead determination by ETAAS after DMSPE separation and preconcentration is summarized in Table 1; the atomization conditions were optimized to ensure reliable quantification of lead across all samples.

Supporting instrumentation included a digital pH meter (OP-211/1, Radelkis, Budapest, Hungary) for pH adjustment, a mechanical shaker (LT3, Nedform, Benešov, Czech Republic) for mixing samples containing TiO_2_, a centrifuge (MPW-360, Mechanika Precyzyjna, Warsaw, Poland) to accelerate phase separation, and an analytical balance (Sartorius 1702, Göttingen, Germany) for precise reagent weighing.

### 2.2. Reagents

All chemicals used were of the highest available purity (p.a., p.a.+, or TraceSELECT^®^ grade). Solutions were prepared using deionized water (Water Pro PS, Labconco, Kansas City, MO, USA). Nanosized titanium dioxide (anatase, <25 nm particle size, >99.7% purity), potassium nitrate, nitric acid, sodium hydroxide, and ammonium dihydrogen phosphate were obtained from Sigma-Aldrich (Steinheim, Germany). To verify the reliability of the proposed extraction procedure, the certified reference material (CRM) TM-25.5, provided by the National Water Research Institute (Burlington, ON, Canada), was analyzed. All glassware was kept in 10% (v/v) HNO_3_ (Lachema, Brno, Czech Republic) for at least 24 h and washed three times with deionized water before use.

### 2.3. Samples

**Figure 1:**
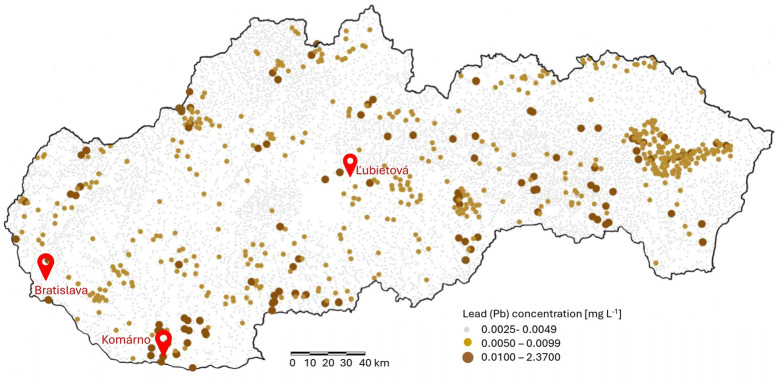
Sampling locations and a simplified map of Slovakia showing lead (Pb) concentrations in surface waters. The map was drawn based on the original online map by Bodiš et al. [9], published as part of the Geochemical Atlas of the Slovak Republic, Part VII: Surface Waters, which displays over 10,000 sampling points.

The developed extraction procedure was applied to the determination of (ultra)trace lead concentrations in three types of real water samples: lake water from Ľubietová (a natural area in the northeastern part of the Slovak Ore Mountains, Inner Western Carpathians, with no significant current industrial development, though historically associated with mining activity), water from a residential well located in the town of Komárno, and wastewater from the treatment plant of a petrochemical facility in Bratislava (Figure 1). Prior to analysis, all samples were filtered through a 0.45 µm membrane filter, acidified with HNO_3_, and stored in polyethylene bottles at 4 °C.

### 2.4. Procedure

As a necessary step prior to ETAAS analysis, the experimental parameters of the DMSPE procedure for lead separation and preconcentration were thoroughly optimized, as summarized in Table 2. The optimization of experimental parameters in this study was carried out using the univariate (one-factor-at-a-time) approach: each parameter—such as pH, ionic strength, TiO_2_ mass, sample volume, and shaking and centrifugation conditions—was individually optimized while keeping the other parameters constant at their previously optimized values. The optimized conditions were subsequently applied to the analysis of real-world samples.

In addition to the experimental parameters for the development of an efficient extraction procedure, the temperature program for reliable lead quantification by the ETAAS method was also optimized. The breakdown of the optimized experimental parameters for ETAAS is given in Table 3. The optimal values for the pyrolysis temperature and the atomization temperature were subsequently used in the calibration as well as in the quantification of lead in model solutions and in real samples after applying the DMSPE procedure.

## 3. Results and Discussion

**Figure 2:**
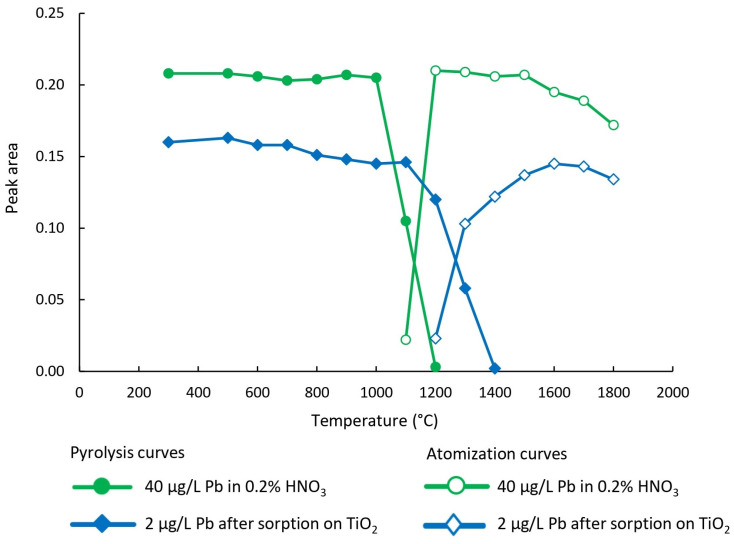
Optimization of the temperature program for ETAAS based on pyrolysis and atomization curves of lead. The figure shows pyrolysis and atomization behavior of lead at different temperatures, comparing standard solutions of lead (40 μg/L in 0.2% (v/v) HNO_3_) with samples containing lead (2 μg/L) adsorbed on TiO_2_. A 2% (w/v) NH_4_H_2_PO_4_ solution served as a matrix modifier in all measurements. The results guided the selection of suitable pyrolysis and atomization temperatures for accurate ETAAS analysis.

The first step toward the reliable determination of the target analyte by ETAAS is the optimization of the temperature program (Figure 2). In this study, primary attention was given to setting the optimal pyrolysis and atomization temperatures, which enabled calibration of the instrument using calibration solutions prepared in 0.2% (v/v) HNO_3_, followed by the analysis of nano-TiO_2_ suspension obtained via DMSPE procedure. These parameters were examined using a lead solution with a concentration of 40 µg/L prepared in 0.2% (v/v) HNO_3_ and a lead solution with a concentration of 2 µg/L, which was used for optimizing the experimental conditions of the DMSPE procedure. In the latter case, slurries containing nano-TiO_2_ with adsorbed lead were injected into the graphite furnace.

The pH of the solution is one of the most critical factors influencing the adsorption of metal ions onto the surface of TiO_2_. Depending on the pH, the surface of titanium dioxide in aqueous media can acquire either a positive or negative charge, as a result of gaining or losing protons through the reactions described by Equations (1) and (2) [10,11]. The point of zero charge (PZC) for TiO_2_, particularly in its anatase form, is typically reported around pH 6.8 ± 0.2 [10,12], though slight variations may occur depending on factors such as surface morphology, synthesis method, and the presence of impurities. This value corresponds to the pH at which the net surface charge density is zero.

In metal oxides, the PZC is typically determined by methods such as potentiometric titration and reflects the pH at which the surface charge becomes independent of ionic strength in the presence of an inert electrolyte. Importantly, a surface charge density of zero does not imply the absence of surface charges, but rather that the positive and negative charges on the surface are balanced [13,14]. The PZC is sometimes referred to as the isoelectric point (IEP), although the two terms are not entirely synonymous (PZC refers to the pH at which the total net surface charge is zero, while IEP refers to the pH at which the zeta potential is zero). For TiO_2_, reported IEP values generally fall in the range of pH 6.2–6.8 [15], and this interchangeable use of PZC and IEP often leads to ambiguity in the literature.

Ti^IV^ − OH + H^+^ ↔ Ti^IV^ − OH^2+^       pH < pH_PZC_ (1)

Ti^IV^ − OH + OH^−^ ↔ Ti^IV^ − O^−^ + H_2_O   pH > pH_PZC_ (2)

When the pH of the solution is above the PZC, the TiO_2_ surface carries a negative charge, which facilitates the electrostatic adsorption of cationic species, such as Pb^2+^. In contrast, under acidic conditions (pH < PZC), the surface becomes positively charged, favoring anion adsorption [16]. However, certain cations, including Pb^2+^, can still be adsorbed under these conditions; Pb^2+^ adsorption is likely favored due to a more energetically beneficial interaction with the surface compared to protons [17]. To suppress such cation adsorption, a very high proton concentration (corresponding to a very low pH) would be required to outcompete Pb^2+^ ions for adsorption sites. At pH values near the PZC, where the TiO_2_ surface is essentially neutral, electrostatic attraction between the surface and lead cations is minimal. Nonetheless, lead adsorption may still occur through non-electrostatic mechanisms, such as specific adsorption (e.g., formation of inner-sphere complexes via direct binding of Pb^2+^ ions to surface hydroxyl groups), surface complexation reactions (involving chemical interactions with deprotonated or partially deprotonated hydroxyl groups), or interactions with the surface hydration layer.

In this study, the effect of sample pH on the extraction of lead cations was investigated within the pH range of 2.0 to 7.0. The extraction efficiency of lead cations increased with pH values from 2 to 4. In the pH range of 4 to 5, fluctuations in extraction efficiency were statistically insignificant, while a slight decrease in efficiency was observed above pH 5. Based on these results, a pH value in the range of 4–5 was selected for subsequent experiments. In addition, the stability of fine suspensions, or slurries, formed from solid substrates like TiO_2_ for direct injection into ETAAS was evaluated to simplify the separation and preconcentration process for (ultra)trace lead determination. The stability of TiO_2_ slurry is highly pH-dependent. At or near the isoelectric point, the surface charge of the sorbent is minimal, promoting nanoparticle agglomeration. In contrast, at pH values above or below the isoelectric point, the increased repulsive forces between particles due to surface charge improve slurry stability. Among the tested pH values, the highest stability for the TiO_2_ slurry was observed at pH 4.5, as evidenced by a model solution of 2 µg/L Pb that was concentrated 20-fold on nano-TiO_2_ and dosed six consecutive times with a relative standard deviation (RSD) of less than 3.5%. This pH value was therefore selected for all subsequent experiments.

**Figure 3:**
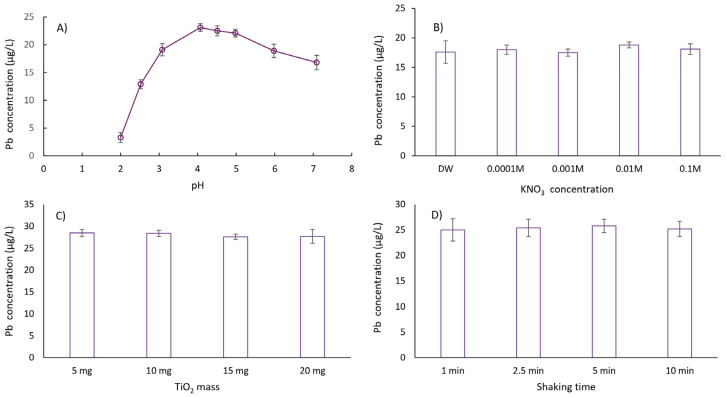
Optimization of the lead extraction procedure using titanium dioxide (TiO_2_) nanoparticles: influence of pH, ionic strength, TiO_2_ mass, and shaking time. This figure illustrates the optimization of a lead extraction procedure using nano-TiO_2_ by examining the influence of four key experimental parameters: (**A**) pH: reflects the acidity level of the solution, up to the neutral point of pH 7.0; (**B**) ionic strength: the concentration of ions in the solution (represented by KNO_3_ concentration); (**C**) mass of TiO_2_: the amount of TiO_2_ nanoparticles used for extraction; (**D**) shaking time with TiO_2_: the duration for which the TiO_2_ nanoparticles are in contact with the lead-containing solution under shaking conditions. Each subplot displays the resulting lead concentration in the solution (µg/L) after the extraction process under varying levels of the specified parameter, while keeping the other parameters constant. The error bars indicate the standard deviation of the method (for *n* = 5). This analysis aims to identify the optimal conditions for maximizing lead removal from the solution using TiO_2_ nanoparticles.

After carefully optimizing all relevant experimental parameters for DMSPE (including ionic strength, modeled by the addition of KNO_3_, mass of nano-TiO_2_, sample volume, nano-TiO_2_ shaking time, centrifugation time and speed, volume of deionized water for slurry preparation, and the optimal preconcentration factor) (Figure 3), the following extraction procedure was established: 10 mg of nano-TiO_2_ was weighed into a 100 mL HDPE container. Then, 50 mL of model solution or sample (pH 4.5 ± 0.1), prepared in 0.01 M KNO_3_, was pipetted into the extraction container containing nano-TiO_2_. The mixture was shaken for 5 min on a mechanical shaker at 22 °C and then centrifuged for 10 min at 4000 rpm. The aqueous phase was decanted by inverting the container. Next, 2.5 mL of deionized water was added to the sorbent, and the mixture was shaken for another 5 min at 22 °C. Before placing the suspension into the autosampler container and injecting it into the graphite atomizer, the suspension was manually mixed for 1 min. Blank samples were prepared using the same procedure, except without the addition of the target analyte.

The volume of 50 mL was selected to achieve a preconcentration factor of 20 using a straightforward and efficient DMSPE procedure with nano-TiO_2_. By employing larger sample volumes, it is possible to attain higher preconcentration factors, which in turn further reduce the LOD after preconcentration and potentially allow detection at “sub-ultra-trace” levels. The use of 50 mL also ensures sufficient interaction between the analyte and the sorbent, which is particularly important when working with (ultra)trace lead concentrations. While this volume could pose a limitation in cases of limited sample availability, it remains practical and easily obtainable for the types of environmental water samples analyzed in this study.

In addition to the main extraction parameters, specific attention was paid to the dosing behavior and background contribution of the TiO_2_ slurry used for direct injection. Direct dosing of the prepared slurry, containing both the sorbent and the adsorbed analyte, offers several advantages over traditional extraction methods. Most notably, it minimizes analyte loss that could occur during the elution step. Moreover, omitting the elution step shortens the overall procedure, making the method more efficient. Despite these benefits, careful optimization of the slurry concentration is essential to ensure reproducible dosing. As the mass of TiO_2_ in the slurry increases, the suspension becomes denser, more prone to settling, and more difficult to dose accurately; therefore, the TiO_2_ mass must be carefully optimized (tailored to the 2.5 mL volume of deionized water employed in this study, Table 2). Blank measurements also require close attention. The “blank” in this context refers to the TiO_2_ suspension prepared in deionized water without any prior lead sorption. Because nano-TiO_2_ itself contains trace levels of lead [18], increasing its mass in the slurry leads to higher lead values in the blank. For this reason, blank values had to be measured precisely and subtracted consistently during each optimization step to ensure accurate analytical results.

Additionally, the potential memory effect after the introduction of TiO_2_ nanoparticles should be considered. In this study, we addressed it by routinely running blank injections between samples, especially those with higher lead concentrations. During repeated analyses of TiO_2_ slurry, particularly over the course of several hundred measurements required for the optimization process, several effects were observed, including residue accumulation, reduced graphite tube lifespan, memory effects, and a gradual decline in analytical accuracy. When non-reproducible results were detected, the graphite tube was replaced.

The reliability of the developed procedure was verified by analyzing CRM TM-25.5, which has a certified lead concentration of 27.0 ± 2.8 µg/L and was diluted 20 times. For this diluted CRM, the DMSPE procedure with a preconcentration factor of 20 was applied. The results are presented in Table 4. The validated DMSPE procedure was then employed to determine (ultra)trace lead concentrations in a lake water sample from Ľubietová, in a water sample from a residential well in Komárno, and in wastewater from a petrochemical facility’s treatment plant. The results are presented in Table 5.

The proposed method—DMSPE using nano-TiO_2_ combined with direct slurry sampling ETAAS—was thoroughly optimized and validated for aqueous matrices, including natural surface water, well water, and industrial wastewater. However, its direct applicability to non-aqueous or more complex sample types, such as biological fluids or soil extracts, remains uncertain without further adjustment and validation. This is because the method relies on the behavior of TiO_2_ in water-based systems, particularly the pH-dependent adsorption of Pb^2+^ ions and the stability of the resulting slurry. While matrix interferences in aqueous systems were effectively controlled through optimized pyrolysis and atomization conditions and the use of a chemical modifier, more complex matrices may present additional challenges. High concentrations of organic matter or dissolved solids could interfere with the sorption efficiency or compromise slurry stability. Furthermore, in buffered or heterogeneous systems, pH adjustment may be more difficult to achieve consistently. Finally, the absence of an elution step, which is an advantage in simple aqueous systems, could permit co-deposition of interfering substances during atomization, thereby affecting signal accuracy and reproducibility. Therefore, while the method shows excellent performance for environmental waters, its applicability to other types of matrices would require further investigation and potentially additional modifications to ensure reliable results.

## 4. Conclusions

In this study, a dispersive micro solid-phase extraction (DMSPE) method using nano-TiO_2_ as a sorbent was successfully developed and optimized for the separation and preconcentration of (ultra)trace concentrations of lead in various types of water samples prior to quantification by electrothermal atomic absorption spectrometry (ETAAS). The procedure employed 10 mg of nano-TiO_2_ for sorption, followed by direct slurry dosing into the graphite atomizer, which eliminated the need for an elution step, thus simplifying the procedure and minimizing analyte loss. Key experimental parameters, including pH, ionic strength, sorbent mass, sample volume, and centrifugation conditions, were carefully optimized to ensure efficient extraction and reliable performance.

The method demonstrated good analytical figures of merit: the optimal preconcentration factor was 20 (determined by the ratio of the 50 mL sample volume to the final slurry volume prepared with 2.5 mL of deionized water), the detection limit (LOD) was 0.11 µg/L, and the quantification limit (LOQ) was 0.34 µg/L. For comparison, without preconcentration, the instrumental LOD and LOQ were 2.31 µg/L and 7.76 µg/L, respectively, highlighting the significant improvement in sensitivity achieved through the application of the DMSPE procedure.

The linear concentration range extended from 0.35 to 3.8 µg/L, with a slope of the calibration curve of 0.0447 (five calibration standards, *n* = 3) and a correlation coefficient greater than 0.997. Repeatability was excellent, with a relative standard deviation (RSD) below 4.5% for 20 replicate model solutions containing 2.0 μg/L of lead. None of the samples contained lead concentrations exceeding the threshold adopted by EU countries, including Slovakia.

The reliability and accuracy of the developed procedure were verified using a certified reference material (CRM TM-25.5), with recoveries ranging from 89% to 95%. The method was further validated through its successful application to real samples of lake water, well water, and industrial wastewater, where recoveries ranged between 92% and 103%. These results confirm that the developed DMSPE-ETAAS method is a sensitive, accurate, and cost-effective approach for monitoring lead contamination in diverse environmental water matrices. Its simplicity, short analysis time, and elimination of organic solvents make it an environmentally friendly alternative to more complex extraction techniques, well suited for routine trace-level lead determination.

## Figures and Tables

**Table 1:** Temperature program for the determination of lead (Pb) by ETAAS.

Step	Temperature (°C)	Ramp (s)	Hold (s)	Ar Flow Rate (mL/min)
Drying	110	1	20	250
Drying	130	5	30	250
Pyrolysis	1000	10	20	250
Atomization	1500	0	5	0
Cleaning	2400	1	2	250

**Table 2:** Optimized DMSPE parameters for lead separation and preconcentration.

Parameter	Tested Range	Optimal Value
pH	2.0–7.0	4.5 ± 0.1
Ionic strength	0–0.1 M KNO_3_	0.01 M KNO_3_
TiO_2_ mass	5–20 mg	10 mg
Sample volume	10–75 mL	50 mL
TiO_2_ shaking time	1–10 min	5 min
Centrifugation time	5–20 min	10 min
Centrifugation speed	2000–4500 rpm	4000 rpm
Deionized water volume	1–5 mL	2.5 mL
Preconcentration factor	10–50	20

**Table 3:** Optimized parameters for lead quantification by ETAAS in the presence of a chemical modifier (2% NH_4_H_2_PO_4_).

Parameter	Tested Range	Optimal Value
Pyrolysis temperature	300–1400 °C	1000 °C
Atomization temperature	1100–1800 °C	1500 °C

**Table 4:** Lead (Pb) concentrations in CRM measured after DMSPE separation and preconcentration.

CRM	Added Pb [µg/L]	Measured Pb ± SD [µg/L]	Recovery [%]
TM-25.5	–	1.28 ± 0.11	95
TM-25.5	1.0	2.24 ± 0.07	89
TM-25.5	2.0	3.21 ± 0.09	93

Certified reference material (CRM) TM-25.5 with a certified concentration of Pb 27.0 ± 2.8 µg/L, diluted 20×; preconcentration factor (PF) of 20 applied; standard deviation (SD) calculated for *n* = 5.

**Table 5:** Lead (Pb) concentrations in real water samples measured after DMSPE separation and preconcentration.

Water Sample	Added Pb [µg/L]	Measured Pb ± SD [µg/L]	Recovery [%]
Well	–	1.28 ± 0.09	–
Well	1.0	2.20 ± 0.10	92
Lake	–	1.76 ± 0.05	–
Lake	1.0	2.74 ± 0.10	98
Wastewater	–	0.36 ± 0.05	–
Wastewater	1.0	1.39 ± 0.07	103

Standard deviation (SD) calculated for *n* = 5.

## Data Availability

The original contributions presented in this study are included in the article. Further inquiries can be directed to the corresponding author.
